# Discerning the Location and Nature of Coke Deposition from Surface to Bulk of Spent Zeolite Catalysts

**DOI:** 10.1038/srep37586

**Published:** 2016-11-23

**Authors:** Arun Devaraj, Murugesan Vijayakumar, Jie Bao, Mond F. Guo, Miroslaw A. Derewinski, Zhijie Xu, Michel J. Gray, Sebastian Prodinger, Karthikeyan K. Ramasamy

**Affiliations:** 1 Physical and Computational Sciences Directorate, Pacific Northwest National Laboratory Richland, Washington 99354, USA; 2 Energy and Environment Directorate, Pacific Northwest National Laboratory, Richland, WA 99354, USA

## Abstract

The formation of carbonaceous deposits (coke) in zeolite pores during catalysis leads to temporary deactivation of catalyst, necessitating regeneration steps, affecting throughput, and resulting in partial permanent loss of catalytic efficiency. Yet, even to date, the coke molecule distribution is quite challenging to study with high spatial resolution from surface to bulk of the catalyst particles at a single particle level. To address this challenge we investigated the coke molecules in HZSM-5 catalyst after ethanol conversion treatment by a combination of C K-edge X-ray absorption spectroscopy (XAS), ^13^C Cross polarization-magic angle spinning nuclear magnetic resonance (CP-MAS NMR) spectroscopy, and atom probe tomography (APT). XAS and NMR highlighted the aromatic character of coke molecules. APT permitted the imaging of the spatial distribution of hydrocarbon molecules located within the pores of spent HZSM-5 catalyst from surface to bulk at a single particle level. ^27^Al NMR results and APT results indicated association of coke molecules with Al enriched regions within the spent HZSM-5 catalyst particles. The experimental results were additionally validated by a level-set–based APT field evaporation model. These results provide a new approach to investigate catalytic deactivation due to hydrocarbon coking or poisoning of zeolites at an unprecedented spatial resolution.

Zeolites are highly crystalline and porous materials possessing a well-defined one- to three-dimensional (1D to 3D) pore structure with diameters between 3 to 12° A^1^. The zeolite materials are built up by tetrahedral metal oxide units having a Si^4+^and Al^3+^ at the center and four oxygen atoms at the corners^2^. More than 100 different types of zeolites are formed based on this primary building block^1^. The substitution of Si^4+^ by Al^3+^ in the zeolite framework forms an ion exchangeable +1 cationic site to maintain the charge balance. These sites are responsible for creating redox, acid, and base properties depending on the nature of the charge-balancing cations^3^. The main parameter that defines the catalytic properties of the zeolites is their aluminum content. The aluminum content can vary from one Al atom per Si atom (zeolite X) to all-silica (silicalite)^2^. The unique combination of the crystalline porous structure along with the catalytic properties of zeolites provides opportunities to use these materials in a wide variety of environmental and catalysis applications. Zeolites play a major role in the petrochemical industry, exploitation of non-conventional petroleum resources (tar sands, heavy crude oil and oil shales), and nonpetroleum resources like gas and coal^4^. Recently, zeolite has been researched heavily in the valorization of biomass for renewable fuel and chemical generation^5^,^6^,^7^,^8^,^9^. It has been particularly investigated for use in the conversion of small oxygenates such as ethanol to hydrocarbons because these compounds can be efficiently generated from biomass resources via thermochemical and/or biochemical pathways^10^,^11^.

It has been reported widely that zeolite deactivation can occur via two main mechanisms. The first one is leaching of alumina (dealumination) from the unit structure of the zeolite in the presence of steam; this mechanism causes the irreversible deactivation of the catalyst^12^,^13^,^14^. The alumina leached from the zeolite unit cell structure remain in the micropores but affect the catalytic activity for the chemistry of interest^1^. Several possible influences relative to the catalyst structure are discussed in the literature, including property modification of Lewis acids^15^, interactions with Brønsted acid sites^16^, replacement of the proton of Brønsted acid sites and neutralization of the acid sites, and the formation of large clusters of amorphous alumina or alumina-silica species that block access to certain micropores^17^. The second mechanism is the formation of bulkier molecules during the transformation of organic compounds in the pores and/or the outer surface and the poisoning of the active sites and/or blocking of the pores. The catalyst deactivation from this mode can be regenerated by burning the catalyst under oxidative conditions at around 550 °C^18^,^19^,^20^. Repeated thermal treatment for spent catalyst regeneration is also shown to cause permanent modification of the zeolite structure and leads to the loss of initial catalyst activity as well as reduced catalyst life time^21^. So identifying the nature and the location of the coking material and changes in Al distribution in catalyst after catalytic reactions becomes imperative for designing a commercially viable catalyst for biomass valorization.

The formation of carbonaceous materials and its effect on the catalytic activity of HZSM-5 in alcohol conversion to hydrocarbon has been extensively studied^21^,^22^,^23^. Currently, the catalyst dissolution technique developed by Guisnet *et al*. is used to identify the nature and the chemical composition of the coking material^8^,^18^, thermogravimetric analysis is used to measure the total coking^5^, and a combination of pore volume measurements and thermogravimetric analysis is used to estimate the coke content of the internal and external surfaces^24^. Occasionally, Focused Ion Beam-Scanning Electron Microscopy-Energy Dispersive X-Ray Spectroscopy (FIB-SEM-EDX) has been used to identify the location of the coke in spent zeolite. More recently, single particle spectroscopic techniques are also being used to study coke formation^25^. Even though such studies have investigated the nature of coke formed during ethanol conversion reactions at a bulk average level or at a macroscopic level, nanoscale imaging of the carbonaceous molecules present in spent catalyst materials using depth profiling is lacking in the literature. Recently, a relatively new characterization capability called atom probe tomography (APT) has been increasingly used to perform 3D quantitative analysis of element distribution in catalyst materials with sub-nanometer–scale spatial resolution and high mass sensitivity up to the few parts per million level of concentration of elements in materials^26^,^27^,^28^,^29^,^30^,^31^,^32^,^33^. It has also been demonstrated that APT can be used to quantitatively image the Al distribution in zeolite materials, and evidence for changes in Al distribution can be obtained by comparing results before and after severe steam treatment of HZSM-5 at 700 °C^34^. Even though the latter study proved the feasibility of using APT for HZSM-5, the severity of steam treatment made it rather difficult to understand the feasibility of studying nanoscale changes in catalyst materials after realistic catalytic conditions under which the combined effect of steam and hydrocarbon coking would exist. More recently work by Schmidt *et al*. analyzed spatial correlation of C_13_ clusters formed in ZSM-5 catalyst particles after 90 minute C_13_ isotopically labeled methanol to hydrocarbon conversion reaction^35^. In their work, based on APT analysis, authors indicated formation of early C_13_ clusters which appeared to correlate with regions of high Al content in ZSM particles. However it is unknown at present if such early stage observations will correlate to longer term reaction times of the order of few days. Also it is unknown how the porous structure of zeolites influence the coke molecule deposition in pores. Additionally a clear analysis of the change in coke distribution from surface to bulk of the ZSM particles is lacking at present. Another aspect of importance is to understand if any molecular signature can be identified by detailed analysis of APT mass-to-charge spectra to possibly develop finger-printing methods for identification of coke molecules and poisoning molecules during catalysis reactions. Therefore, in the study reported here we used a multimodal approach including C K-edge x-ray absorption at beam line 6.3.2 of the Advanced Light Source (ALS) facility at Lawrence Berkeley National Laboratory, along with ^13^C cross polarization magic angle spinning nuclear magnetic resonance (CP-MAS NMR) and APT to investigate element distribution within a HZSM-5 catalyst particle with a Si-to-Al ratio of 35 in an as-synthesized condition and after ethanol conversion reaction at 350 °C for 72 hours at which point a combined effect of steam and coke formation exists.

## Results and Discussion

**Figure 1:**
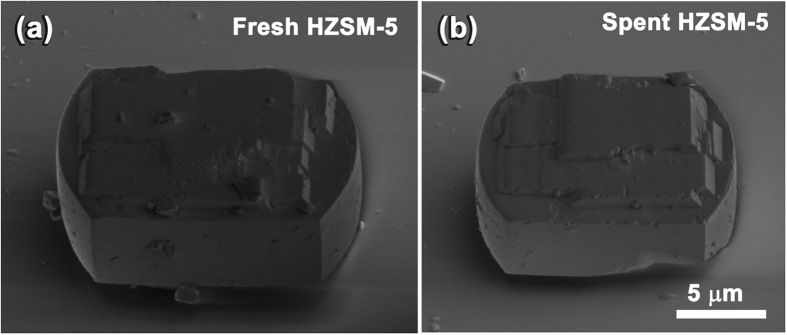
Scanning electron microscopy secondary electron images of (**a**) fresh and (**b**) spent HZSM-5 particles.

**Figure 2:**
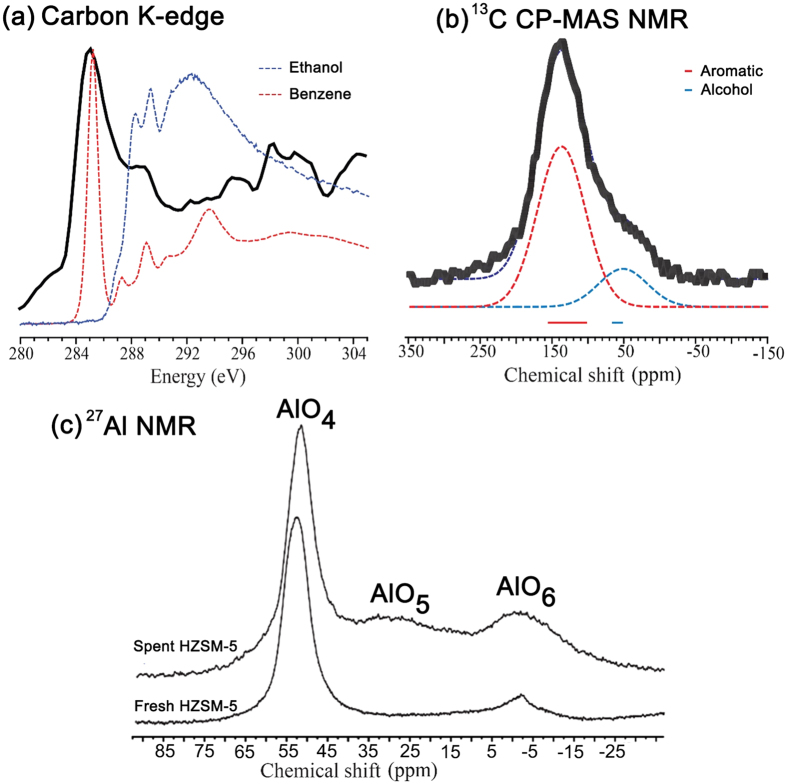
(**a**) The C K-edge XAS spectrum obtained from a 72-hour ethanol-treated HZSM-5 sample along with benzene (red) and ethanol (blue) gas phase reference XAS spectra. The reference XAS spectra were obtained from the following gas phase core excitation database: http://unicorn.mcmaster.ca/corex/cedb-title.html. (**b**) ^13^C CP-MAS NMR spectra of a 72-hour ethanol-treated HZSM-5 sample measured at 11.7 Tesla magnetic field with a spinning speed of 10 kHz. The deconvoluted spectra (dotted lines) and typical chemical shifts of aromatic (red) and alcohol (blue) components are highlighted. (**c**) 27Al NMR of fresh and spent HZSM catalyst measured at 11.7 T magnetic field under magic angle spinning speed of 14 kHz.

Scanning electron microscopy (SEM) images of fresh and ethanol-reacted HZSM-5 catalysts are provided in Fig. 1. The images show no substantial change in the catalyst particle morphology between fresh and spent catalyst. The pristine white powder turned black after catalysis, indicating the presence of carbonaceous coke materials within the pores. C K-edge X-ray absorption near edge structure (XANES) spectra of spent HZSM-5 after 72 hours of ethanol treatment are given in Fig. 2(a) along with benzene and ethanol gas phase reference spectra obtained from the core excitation database (http://unicorn.mcmaster.ca/corex/cedb-title.html). The main absorption peak (~284.5 eV) of spent catalyst clearly matches the benzene reference spectra indicating the presence of aromatic type compounds as part of the coke residuals. However, differentiating the residual parent ethanol is more complex because of the possible overlap of its main absorption peak (~288.5 eV) with aromatic secondary peaks. Hence, ^13^C CP-MAS NMR measurements were carried out to distinguish the coke molecules (as aromatic compounds) from the residual parent ethanol molecules. Two broad lines representing the typical chemical shift of aromatic compounds (100–160 ppm) and alcohol compounds (50–70 ppm) were clearly identified. The combined XANES and NMR measurements reveal that the carbonaceous coke material causing the black color of the spent catalysts is mainly composed of aromatic compounds and that parent ethanol occurs as a residual component. Thermogravimetric analysis (temperature-programmed oxidation) of the spent catalyst shows the coke content is about 6.5 wt % of the total weight of the catalyst (see further in formation in Figure S2).

The 27Al MAS NMR of fresh HZSM-5 catalyst shows predominantly tetrahedral alumina (AlO4) site along with small concentration (<5%) of octahedral alumina (AlO6) site (see Fig. 2(c)) in accordance with previous reports^36^,^37^. Spent catalyst registers two broad peaks indicating increased octahedral alumina sites (~15%) and penta-coordinated alumina (~8%) relative to fresh catalyst. The emergence of new alumina sites with heavily broadened NMR linewidth indicates aluminum structural distortion at the pores due to the formation of coke products. In particular, the hydrocarbon and aromatic carbon based molecules can chemically interact with Bronsted acid proton in the vicinity of the Al leading co-ordination change as well as structural distortion observed in the NMR spectra. This result suggests that the structural modification of the zeolite by dealumination process due to the coke formation during catalyst process. Such dealumination of zeolite H-ZSM-5 has been observed during the catalytic process of different oxygenates (i.e., methanol, ethanol and bio-oil) to hydrocarbons, which is attributed to steaming at high reaction temperatures^38^. In addition, the dominant tetrahedral alumina peak also shifts towards higher field (i.e. ~3 ppm towards lower chemical shift) in the spent catalyst. As the pore is filled with coke product involving aromatic and hydrocarbon, the water content is reduced and the Bronsted acid proton spends more time in the vicinity of the AI leading to high field chemical shift observed in 27Al NMR of spent catalyst^36^,^37^.

**Figure 3:**
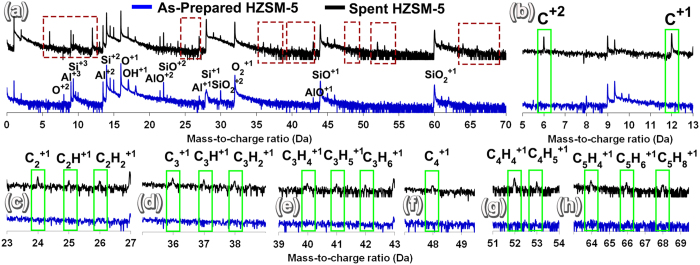
(**a**) Mass-to-charge spectra comparison of as-prepared and spent HZSM-5 (**b–h**), showing the identification of different carbon species detected in spent HZSM-5 when compared with fresh HZSM-5.

APT specimens were prepared from the fresh and spent HZSM-5 particles and analyzed to understand the distribution of individual elements. Before comparing the APT results of 3D elemental distribution in fresh and spent HZSM-5, the mass-to-charge spectra of as-prepared HZSM-5 (blue) and spent HZSM-5 (black) were compared (Fig. 3). Both as-prepared and spent HZSM-5 mass-to-charge spectra indicated elemental peaks of Si^+1, +2, +3^, Al^+1, +2, +3^, and O^+1^ and molecular peaks of O_2_^+1^, OH^+1^, SiO^+1, +2^, AlO^+1, +2^, and SiO_2_^+1, +2^. However, a complete absence of any carbon elemental peaks was noted in the fresh HZSM-5 mass-to-charge spectra, while clear evidence of strong C^+1^ and C^+2^ peaks was observed in spent HZSM-5 (Fig. 3(b)). Many C-H molecular peaks were also only uniquely observed in spent HZSM-5, as shown in Fig. 3(c–h). The presence of C_2_^+1^, C_2_H^+1^, C_2_H_2_^+1^, C_3_^+1^, C_3_H^+1^, C_3_H_2_^+1^, C_3_H_4_^+1^, C_3_H_5_^+1^, C_3_H_6_^+1^, C_4_^+1^, C_4_H_4_^+1^, C_4_H_5_^+1^, C_5_H_4_^+1^, C_5_H_6_^+1^ and C_5_H_8_^+1^ were noted in spent HZSM-5.

**Figure 4:**
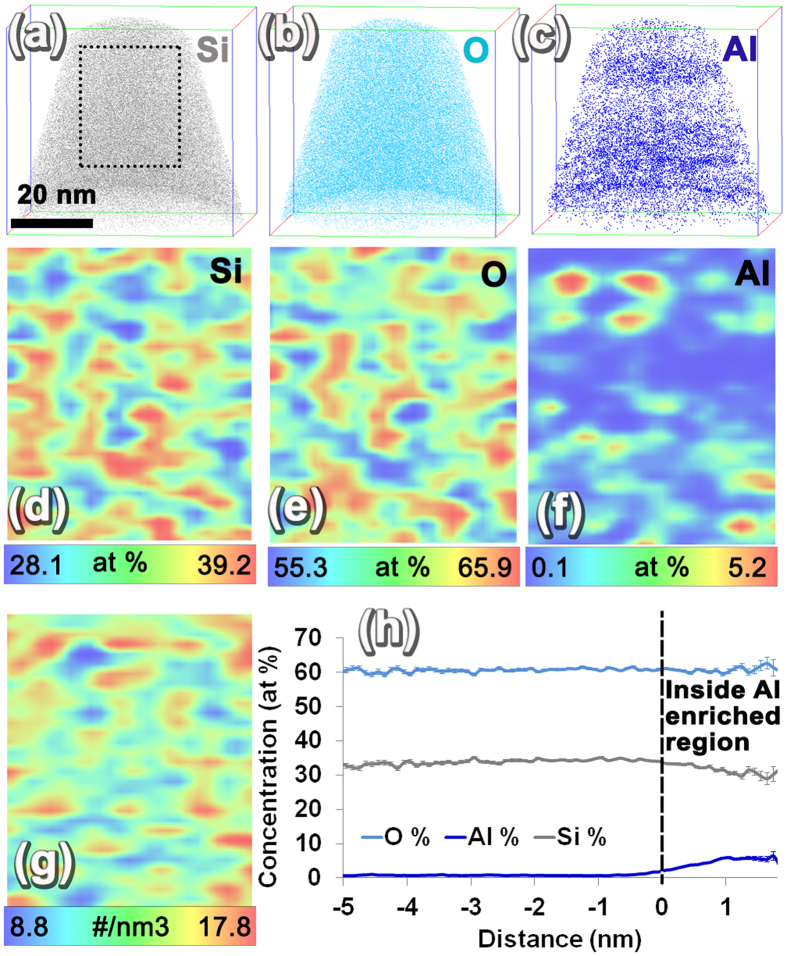
Fresh HZSM-5 APT results. (**a–c**) Ion maps of Si, O, and Al; (**d–f**) a 2D composition map across a 1 nm × 25 nm × 30 nm volume slice of Si, O, and Al; (**g**) a 2D ion density map; and (**h**) a proximity histogram across 3 at% Al iso-concentration surfaces.

The 3D ion distribution map of Si (grey), O (light blue), and Al (dark blue) from fresh HZSM-5 is given in Fig. 4(a-c). The APT reconstructed volume corresponds to 58.58 nm × 57.73 nm × 84.88 nm close to the top surface of the HZSM-5 particle. From these images clear evidence of rather non-uniform distribution of Al can be seen (Fig. 4(c)). To better understand the non-uniformity of element distribution 2D composition maps were plotted using a 1 nm × 25 nm × 30 nm volume slice perpendicular to the APT result (at the location shown by the dotted rectangle in Fig. 4(a)). The 2D composition map of Si, O, and Al are given in Fig. 4(d–f) in which dark red corresponds to the higher concentration of the elements and dark blue represents the lower range of concentration. The color scale bar showing the concentration variation for each element in atomic percent is given below each image. For these 2D concentration maps, both elemental and decomposed contributions of molecular ions were used. From the 2D maps, it is very clear that a somewhat periodic non-uniformity of Si and O exists throughout the HZSM-5, and Al is non-uniformly distributed without this periodicity forming what appears to be isolated sparsely distributed regions of Al enrichment in the reconstruction. The variation in major elements, i.e., Si and O distribution, can be influenced by a number of factors, the first of which is the porosity in the HZSM-5, which is on the order of 5.4–5.6 angstroms^1^. However, the capability of visualizing the porosity in zeolites using APT is still not proven conclusively, because of the occurrence of local magnification, which can lead to trajectory crossing during the evaporation of the needle-shaped specimen of a material that has embedded pores^39^,^40^. Nonetheless, past efforts have attempted to locate voids in irradiated structural materials using iso-density surfaces in APT data^41^,^42^,^43^. In line with such efforts, a 2D map of all ion density was plotted in which a variation in ion density is also clearly visible where the ion density oscillates between higher and lower values rather periodically (Fig. 4(g)). This may be an indication of the periodic pores in HZSM-5, and the validity of this attribution is tested by computational simulation of APT evaporation and reconstruction of a porous material in the latter part of this manuscript. The Al distribution in the 2D map (Fig. 4(f)), however, looks distinctly different than the Si and O 2D maps (Fig. 4(d–e)). The compositional partitioning between the Al-rich regions and matrix was measured by calculating a proximity histogram^44^ composition profile across all of the regions that had greater than 3 at% Al within the reconstruction (Fig. 4(h)). In accordance with the proximity histogram, inside the Al-rich regions the concentration of Al reached as high as 5.42 ± 0.91 at% Al.

**Figure 5:**
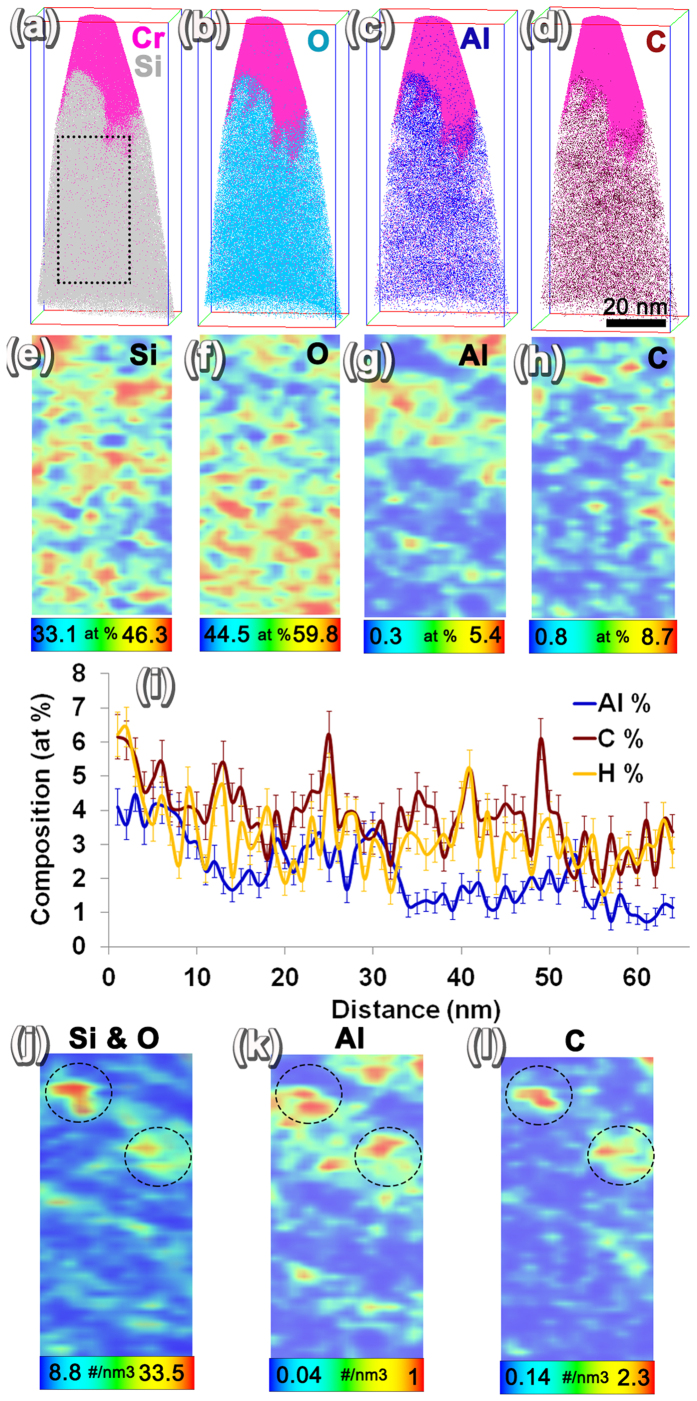
APT results of spent HZSM-5. Ion maps showing the Cr cap in pink along with (**a**) Si, (**b**) O, (**c**) Al, and (**d**) C. 2D ion maps plotted using a 1 nm × 25 nm × 50 nm slice of the APT reconstruction show the (**e**) Si, (**f**) O, (**g**) Al, and (**h**) C distribution. The scale bar is given below each image to show the concentrations corresponding to maximum and minimum composition values. (**i**) 1D concentration profile from the surface of the HZSM-5 to the bulk plotted using a 10 nm diameter cylinder showing changes in average Al, C, and H concentration. The ion densities of (**j**) Si and O, (**k**) Al, and (**l**) C are compared showing the spatial correlation.

APT results from spent HZSM-5 after the ethanol conversion to hydrocarbon reaction for 72 hours are shown in Fig. 5. The total volume analyzed by APT is 49.91 nm × 46.69 nm × 109.93 nm. A 100 nm thick Cr capping layer was deposited on the surface of the catalyst particles to protect the top surface of the spent catalyst material before the start of APT sample preparation. The capping layer was not removed entirely during annular milling so that the APT analysis could start from the capping layer, proceed to the HZSM-5 top surface, and then to the HZSM-5 bulk. Hence, the Fig. 5(a–d) ion maps show the Cr capping layer in pink. The interface between the top surface of HZSM-5 and Cr capping layer was irregular, presumably because of the irregular surface shapes visible in the SEM images in Fig. 1. The distributions of Si, O, Al, and C in the HZSM-5 are shown in the composite maps of these elements with Cr capping in Fig. 5(a–d). Two clear observations can be made based on the ion maps: the first is a non-uniform distribution of Al and the second is a distinct clustering of carbon. To understand element distribution with better clarity, 2D concentration maps were plotted using a 1 nm × 25 nm × 50 nm volume slice of the APT reconstruction at the location shown by the dotted rectangle in Fig. 5(a). The 2D maps are provided in Fig. 5(e–h) and a periodic non-uniformity in Si and O distribution is observable, similar to the as-prepared HZSM-5 APT results (Fig. 4(d–e)). In addition, clear evidence for non-uniform distribution of Al and C can also be observed in Fig. 5(g–h). Both elemental and decomposed molecular ion contributions were considered to derive the 2D concentration maps. Thereafter, in order to measure the composition variation from the top surface of HZSM-5 to the bulk, a composition profile was plotted using a 10 nm diameter cylindrical region from the top surface of the HZSM-5 to the bulk (Fig. 5(i)). Very clear evidence for change in the average Al concentration from about 4 at% closer to the surface to about 1.5 at% in the bulk of HZSM-5 was observed. Similarly, C concentration was observed to drop from about 6 at% closer to the surface to 3 at% in the bulk. For a clearer understanding of the spatial correlation between the ion density of Si and O with Al and C, 2D ion density maps were plotted using the same 1 nm × 25 nm × 50 nm volume as shown in Fig. 5(j–l). From the 2D ion density maps a spatial correlation of the regions of high density of C with similar high densities of Si, O, and Al is observed, especially in the regions highlighted by the black dashed line circle. Such spatial correlations in ion density maps of APT reconstruction can be indicative of the presence of pores in that vicinity. This attribution is also tested by simulating the APT evaporation and reconstruction of a material with pores filled with a high evaporation field material.

To further evaluate the validity of the experimental APT results, a level-set–based field evaporation model was developed for porous materials; it included empty pores as well as pores filled with a high evaporation field material. A 2D simulation domain with 192 (x) × 96 (y) nm was used, as shown in Fig. S3. The voltage between the bottom (y = 0 nm) and the counter electrode (y = 96 nm) was given as 5808 V. The specimen was placed at x = 0 nm with a width of 48 nm, and a height of 40 nm with a round tip of radius 24 nm. The pore diameter was taken to be 8 nm, and the distance between pores was kept at 9.6 nm for both x and y directions, as shown in Fig. 1. The detector was 10 cm away from the specimens, and the positions where the evaporated ions hit on the detector were recorded and used for the reconstruction. The specimen shape evolution was simulated using the level-set method. The trajectories of ions were calculated by using the first principle of dynamics. The details about the simulation models, numerical scheme, and coupling of the specimen shape evolution simulation and the trajectories calculation were introduced in our previous work^45^,^46^. The reconstruction in this study followed Bas *et al*.’s^47^ first and the second method, which were also used to develop the experimental APT data reconstructions. Two test cases were studied: a first case of unfilled pores, and another one where the pores were filled by a high field material with twice the evaporation field of the parent matrix material. For the simulations, evaporation strengths of 26.7 V/nm for the matrix material and 53.4 V/nm for the high evaporation field material in the pores were assumed. The relative permittivity for both materials was assumed to be 18. The K factor for reconstruction was kept at 3.3, and the image compression factor was taken to be 1.65.

**Figure 6:**
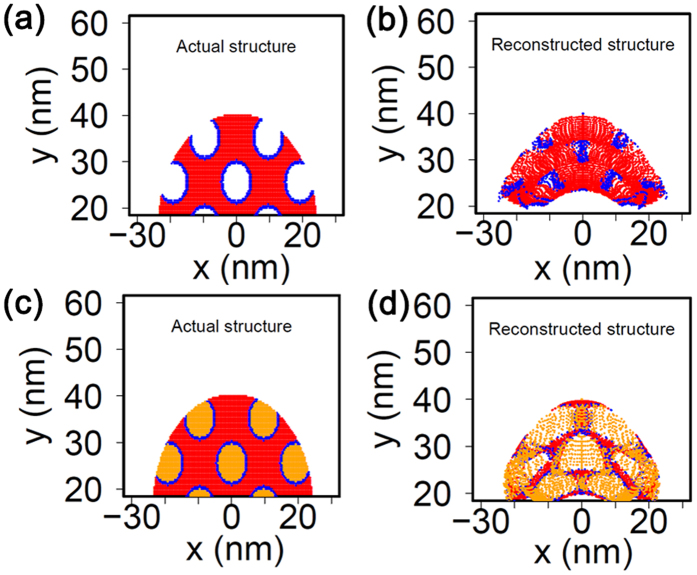
Level-set simulation. (**a**) framework for porous material, (**b**) the reconstructed simulated APT result showing the non-uniform distribution of ions, (**c**) the simulation framework for the same material with pores filled with a high electric field material, and (**d**) the reconstruction from the material with filled pores.

The reconstruction results in Fig. 6(a) show the simulated geometry of material with unfilled pores where the atoms at the periphery of pores are highlighted in blue. The reconstructed simulated APT data of the same material are shown in Fig. 6(b) in which considerable presence of edge atoms inside the pores is observable. This can be attributed to trajectory crossing events during evaporation of porous material^39^. In spite of the local distortions in ion trajectory, based on Fig. 6(b) it is clear that there can be a non-uniform density of atom distribution in the location of pores.

Next, a case with pores filled with a material with double the evaporation field strength of the parent material was considered, as shown in Fig. 6(c) in which the yellow color denotes the high field material within the pores. The reconstruction of the simulated APT result is shown in Fig. 6(d), which clearly also shows the high field material to be confined inside the pore regions in the APT reconstructions. By comparing these simulation results with the experimental results, it can be concluded that one of the main causes of the non-uniform ion density is the presence of pores and the presence of material inside the pores. Note that in the simulation a single evaporation field was assumed for all of the ions from the matrix, but in reality, because of the evaporation of complex ions that presumably have a slightly different evaporation field, the experimental density distribution can have additional contributions including those from evaporation of molecular ions, which will be the subject of a separate future study.

Thus, upon comparing experimental results from the as-prepared HZSM-5 particle and spent HZSM-5 particle with level-set simulation results, a few conclusive observations were derived. 1) A comparable periodic non-uniform distribution of the major elements of HZSM-5 (Si and O) is seen under both conditions. 2) Isolated regions of Al enrichment are seen under both conditions and they look distinctly different than the Si and O distribution. 3) Carbonaceous peaks are only observed in the mass-to-charge spectra of spent HZSM-5, and both elemental C and molecular C-H species are observed. 4) Carbonaceous species appear to aggregate as clusters, possibly within the pores of spent HZSM-5. Based on the observed variation in the ion density and concentration of Si and O in as-prepared HZSM-5, it is possible that APT is capturing the indication of the presence of pores. This result is the first evidence of non-uniform ion density in APT results for a porous oxide material.

The Al distribution appears to be independent of the periodic variation in Si and O in both as-prepared and spent HZSM-5. In recent work by Perea *et al*., using APT the Al distribution in as-synthesized and severely steam-treated (at 700 °C) HZSM-5 with a Si/Al ratio of 17 was compared. Through their studies, a non-uniform distribution of Al was seen both in as-fabricated and severely steam-treated HZSM-5, as revealed by the Pearson’s coefficient values of 0.7 and 1 for the Al-Al nearest neighbor distributions in APT data^34^. In that study, by steaming HZSM-5 at a higher temperature of 700 °C, the Al-Al nearest neighbor distribution became more non-uniform^34^. Compared to the work by Perea *et al*., the HZSM-5 used in this study had a Si/Al ratio of 35, implying a much lower amount of Al concentration and ethanol treatment temperature of 350 °C at which steam and hydrocarbons interact with the HZSM-5 catalyst in an industrially relevant catalytic condition. In another recent work by J. E. Schmidt *et al*. C_13_ clusters were noted to be present in low Si/Al ratio regions (or high Al concentration regions) after 90 minutes of methanol-hydrocarbon conversion reaction^35^. Based on our APT results, we also observed a non-uniform distribution of Al in as-prepared HZSM-5 as revealed by the 2D ion concentration map in Fig. 4(f), which shows a variation of concentration of 0.1–5.2 at%. A comparable degree of non-uniformity in Al distribution was also observed in the spent HZSM-5 after ethanol conversion reaction at 350 °C based on a 0.3–5.4 at% concentration variation in the 2D concentration maps Fig. 5(g).

The organic carbonaceous molecules present in spent HZSM-5 pores after ethanol conversion reaction were identified to be predominantly aromatic compounds along with some residual alcohol compounds based on the combination of C K-edge XAS and ^13^C CP-MAS NMR spectroscopy. Based on thermogravimetric analysis, the spent catalyst was demonstrated to have a coke content of 6.5 wt% of the total weight of catalyst. These carbonaceous molecules can either ionize and field evaporate directly during APT experimentation or they can fragment to form smaller molecular fragments and then ionize to contribute to the final APT mass-to-charge spectra from the spent HZSM-5. The absence of any molecular signatures with higher than 5 C atoms in the observed mass-to-charge spectra most conclusively implies a fragmentation mechanism operating during APT. At present, a number of investigations are ongoing to identify the mechanism of the ionization of organic molecules during laser-assisted APT, which is focused on distinguishing the direct ionization or molecular fragmentation process^48^. However, it can be conclusively stated that the elemental peaks of C and molecular species of C and H observed in APT mass-to-charge spectra originated from the carbonaceous molecules trapped in zeolite pores, thereby providing the clear demonstration of a spatially resolved mapping of carbonaceous molecules in a spent HZSM-5 catalyst material after being exposed to realistic catalytic conditions. Based on this demonstration, we believe detailed investigations can be carried out with different catalytic reactions on HZSM-5 catalysts to clearly study the finger-printing of coke molecules formed inside HZSM-5 pores using systematic APT investigations combined with other conventional coke characterization methods.

## Conclusion

With these multimodal characterization and modelling results, it is very clear that the as-synthesized HZSM-5 particles with a Si/Al ratio of 35 have a non-uniform distribution of Al that is also present after ethanol conversion reactions. On comparing 27Al MAS NMR spectra of fresh and spent HZSM-5, coke induced local structural changes of Al within the frame work structure. C K-edge XAS and ^13^C-MAS-NMR analysis revealed the aromatic nature of the coke molecules in the spent catalyst. From APT results, clear evidence of elemental C, molecular C, and C-H complexes was observed to exist in spent HZSM-5 catalyst particles, which are fragmentation products of coke molecules in spent catalyst. These carbonaceous components were observed to aggregate in the form of small carbonaceous clusters within the spent HZSM-5 particles. A clear change in the composition of C and Al from the surface to the bulk of the spent HZSM-5 catalyst was also observed. This is direct evidence of the hydrocarbon molecules present in the pores of HZSM-5 catalyst particles after catalytic operation. Such carbonaceous molecules are known to lead to temporary deactivation of HZSM-5 catalyst during extended catalysis reactions necessitating a regeneration process to burn off the agglomerated carbonaceous compounds. Thus, by using APT we have conclusively showed evidence of non-uniform elemental distribution in HZSM-5 catalyst during realistic catalytic conditions and provided atomic-scale imaging evidence of the observation of hydrocarbon molecules within the pores in HZSM-5 catalyst after ethanol conversion reactions. Therefore, we anticipate APT to be a unique method for understanding coke formation or other poisoning mechanisms operating during hydrocarbon conversion reactions, thereby permitting unprecedented understanding of catalytic deactivation mechanisms that are highly relevant for important efforts to produce renewable fuels and chemicals from biomass.

## Methods

### Material Synthesis

HZSM-5 with a Si-to-Al ratio of 35 was synthesized following the hydrothermal procedure using tetrapropylammonium (TPA) ions as the template^49^. The chemicals used in the synthesis were sodium silicate (27% SiO_2_, 14% NaOH), aluminum sulfate, sodium chloride, sulfuric acid, and tetrapropylammonium bromide (in-house laboratory synthesized). The molar ratios for the hydrogel were SiO_2_/Al_2_O_3_ = 90.4; OH^−^/SiO_2_ = 0.49; H_2_O/OH^−^ = 42.2; NaCl/Al_2_O_3_ = 85.9; and tetrapropylammonium bromide/Al_2_O_3_ = 9. The gel was kept for 10 hours at ambient temperature, and sulfuric acid was added subsequently to decrease the pH of the gel (OH^−^/SiO_2_ = 0.19) prior to crystallization. The synthesis was carried out at 120 °C for 24 hours and subsequently at 160 °C for 5 hours. The solid product was recovered by filtration, washed with deionized water, and dried at 100 °C overnight. First, the sodium form of the ZSM-5 material was ion exchanged with 0.1 M NH_4_NO_3_ solution at 80 °C for 2 hours to convert it to the NH_4_-form. After 2 hours, the solution was centrifuged and the filtrate was washed with deionized water several times. The whole procedure was then repeated twice. The NH_4_-form was then calcined at 450 °C in air for 6 hours with a heating rate of 5 °C/min to decompose and remove the ammonia to yield the activated H-form of the ZSM-5. The XRD results from as-synthesized, calcined, NH4-form and H-form of ZSM-5 is shown in supplementary figure 4. The ^27^Al-NMR analysis of as-synthesized, calcined, NH4-form and H-form of ZSM-5 are shown in supplementary figure 6 revealing predominantly tetrahedral Al in all the conditions.

### Ethanol Conversion Treatment

The ethanol conversion treatment of the synthesized HZSM-5 catalysts was carried out in a continuous fixed-bed, down-flow 3/8-inch stainless steel tube reactor. The catalyst treatments were conducted at 350 °C and at atmospheric pressures at an ∼12.5 mL/min nitrogen (N_2_) carrier gas flow rate. The catalyst was packed in the middle of the reactor tube between thin layers of glass wool in an isothermal zone. The weight hourly space velocity of 2.85 h^−1^ for the ethanol treatment was maintained. The treatment was conducted for 72 hours; then the spent HZSM-5 sample was collected for further analysis. At these operating conditions the ethanol went through intramolecular dehydration to form ethylene and water in a single step. Then, ethylene went through a complex network of reaction pathways such as oligomerization, dehydrocylization, and hydrogenation to form a mixture of gasoline-range hydrocarbons^5^,^23^. Throughout the 72-hour experiment ethanol conversion was 100 percent as shown in supplement figure S1. For the first 8 hours the product composition contained a mixture of alkylated aromatics, alkanes, and alkenes between the C_2_ and C_10_ numbers. Beyond 8 hours the hydrocarbon product primarily consisted of ethylene. The ethanol conversion and the product carbon selectivity information can also be found in the Supplementary figure 1. The comparison of XRD spectra from fresh and spent HZSM-5 is shown in supplementary figure 5.

### Characterization

Temperature-programmed oxidation-thermogravimetric analysis (TPO-TGA) was carried out by heating a measured amount of spent catalyst to 800 °C at a heating rate of 5 °C/min in a gas flow of 5 vol% oxygen (O_2_) in N_2_ at 10 mL/min.

The ^27^Al single pulse MAS NMR measurements were carried out using Varian 500 Inova spectrometer (B_0_ = 11.7 T; 27Al Larmor Frequency is 130.43 MHz). The ^13^C NMR measurements with proton decoupling were done using a Varian 300 Inova spectrometer (B_0_ = 7.04 T; 13 C Larmor Frequency is 75.43 MHz). All the NMR measurements were carried out using 4 mm standard NMR rotor with a spinning speed of 10 kHz under room temperature (~24 °C) conditions. The observed ^27^Al and ^13^C chemical shifts were externally referenced (σ_iso_ = 0 ppm) to a 1 M aqueous aluminum chloride and pure tetramethylsilane solutions respectively.

X-ray powder diffraction (XRD) patterns of all HZSM-5 samples were recorded on a Phillips X-Pert (50 kV and 40 mA) diffractometer using Cu Kα radiation (λ¼ 1.54059 Ǻ). Each sample was scanned in the range between 10° and 80°.

C K-edge XAS spectra were collected for fresh and spent HZSM-5 powder samples at beamline 6.3.2 in the ALS facility.

The fresh and spent HZSM-5 particles were dry dispersed on a clean Si wafer and coated with 100 nm thick Cr film using an ion beam sputter coating system before APT sample preparation. The SEM imaging and APT specimen preparation were conducted using an FEI Helios Nanolab 600 dual-beam FIB system. The FIB lift-out procedure was used to extract a cantilever of the HZSM-5 particles and it was then mounted on Silicon micro-tip arrays and sharpened by annular milling to achieve a needle-shaped specimen that had an end diameter of less than 100 nm. These specimens were analyzed using a CAMECA LEAP4000XHR APT system and a 355 nm ultraviolet laser with 100–200 pJ laser pulse energy, 40–60 K specimen temperature, and 200 KHz pulse repetition rate. The APT results were analyzed using IVAS 3.6.8 software.

## Additional Information

**How to cite this article**: Devaraj, A. *et al*. Discerning the Location and Nature of Coke Deposition from Surface to Bulk of Spent Zeolite Catalysts. *Sci. Rep.* **6**, 37586; doi: 10.1038/srep37586 (2016).

**Publisher’s note:** Springer Nature remains neutral with regard to jurisdictional claims in published maps and institutional affiliations.

## Supplementary Material

Supplementary Information
