# Supplementary material for: Discerning the Location and Nature of Coke Deposition from Surface to Bulk of Spent Zeolite Catalysts
Source: Sci Rep. 2016 Nov 23;6:37586. doi: 10.1038/srep37586 (PMC5120296; doi:10.1038/srep37586)
Supplement: Supplementary Information [file srep37586-s1.pdf]

SUPPLEMENTARY INFORMATION

Discerning the Location and Nature of Coke Deposition from Surface to Bulk of Spent Zeolite Catalysts

Arun Devaraj [a,*], Murugesan Vijayakumar [b], Jie Bao [b], Mond F. Guo [b], Mirosław A. Derewinski [a], Zhijie Xu [a] Michel J. Gray [b], Sebastian Prodinger [a], Karthikeyan K. Ramasamy [b,*]

[a] Physical and Computational Sciences Directorate, Pacific Northwest National Laboratory Richland, Washington 99354, USA
[b] Energy and Environment Directorate, Pacific Northwest National Laboratory, Richland, WA 99354, USA
Corresponding authors: arun.devaraj@pnnl.gov, karthi@pnnl.gov

SUPPLEMENTARY INFORMATION

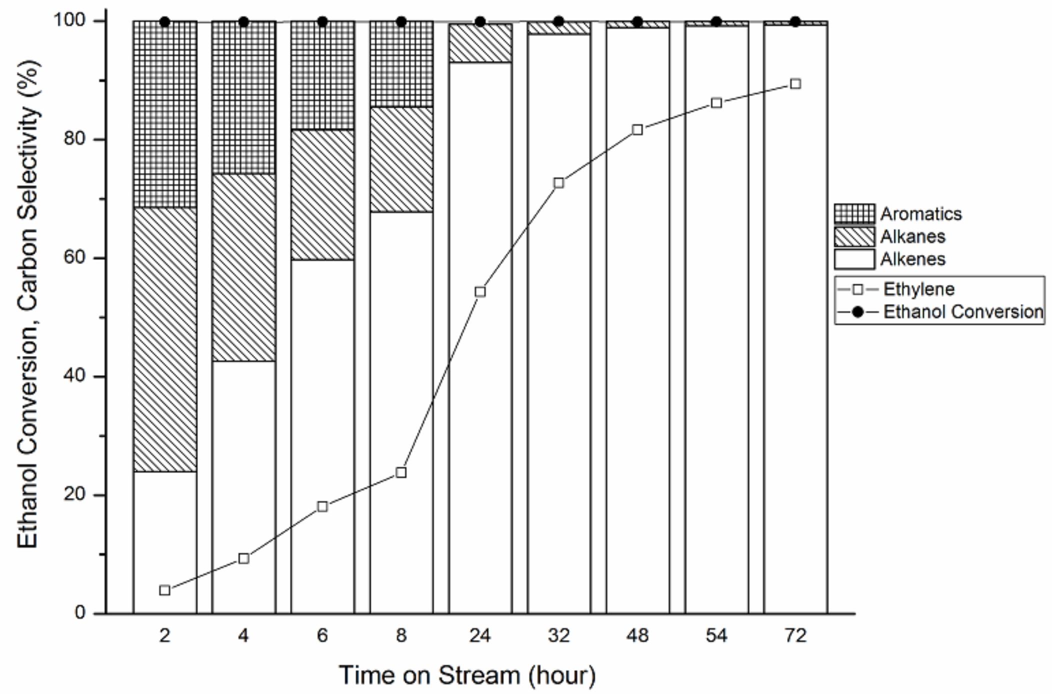

Supplementary Figure 1: Ethanol conversion and the product carbon selectivity over the HZSM-5 catalyst at 350°C, atmospheric pressure and 2.9h^{-1} weight hourly space velocity.

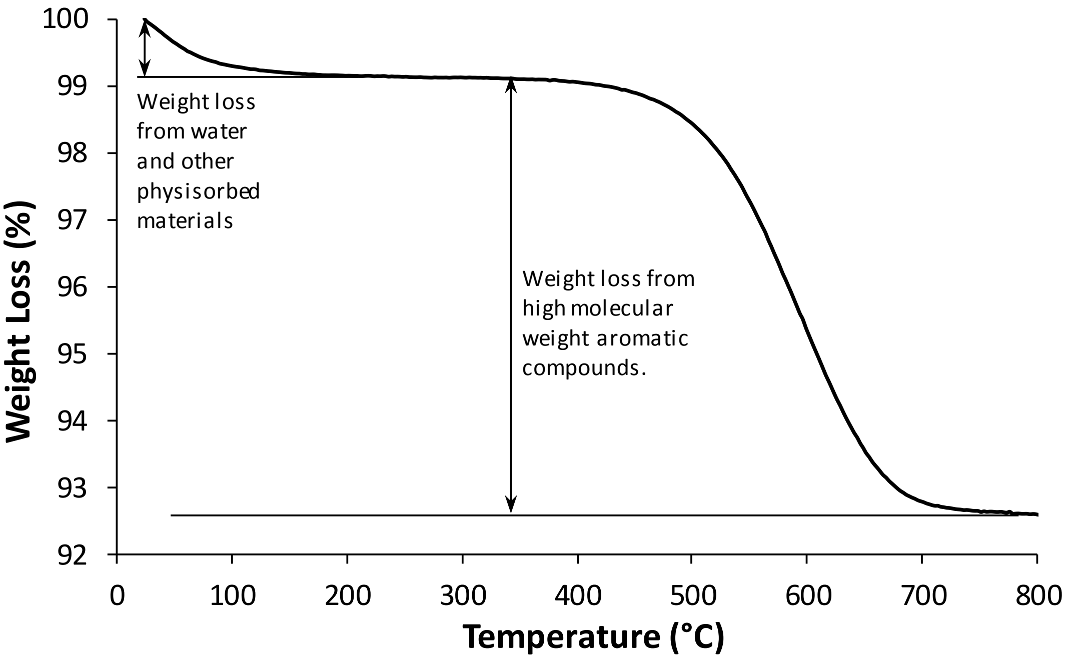

Supplementary Figure 2: Spent catalyst weight loss from the TPO-TGA using 5vol% O_2 in N_2 carrier gas with 5°C/ min ramp rate to 800°C from room temperature.

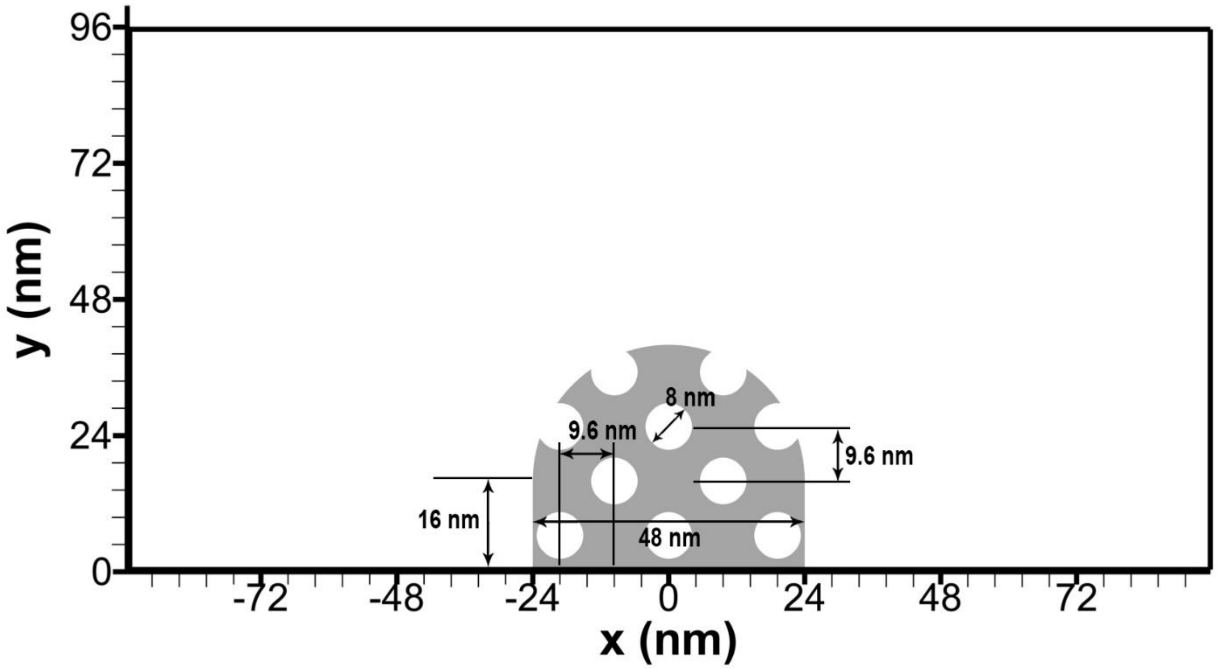

Supplementary Figure 3: The APT simulation domain and the specimen setup.

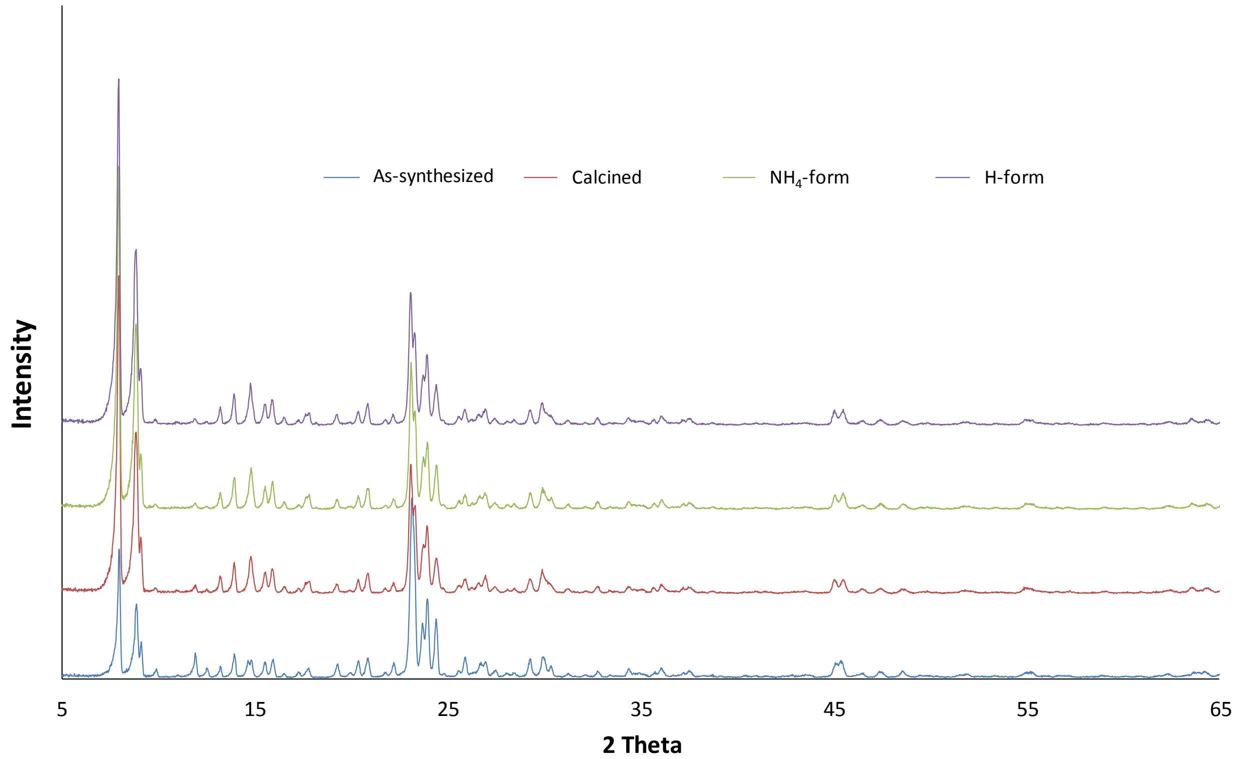

Supplementary Figure 4: XRD profile of the zeolite material tested in the form of as-synthesized, calcined, NH_4 form, and H-form.

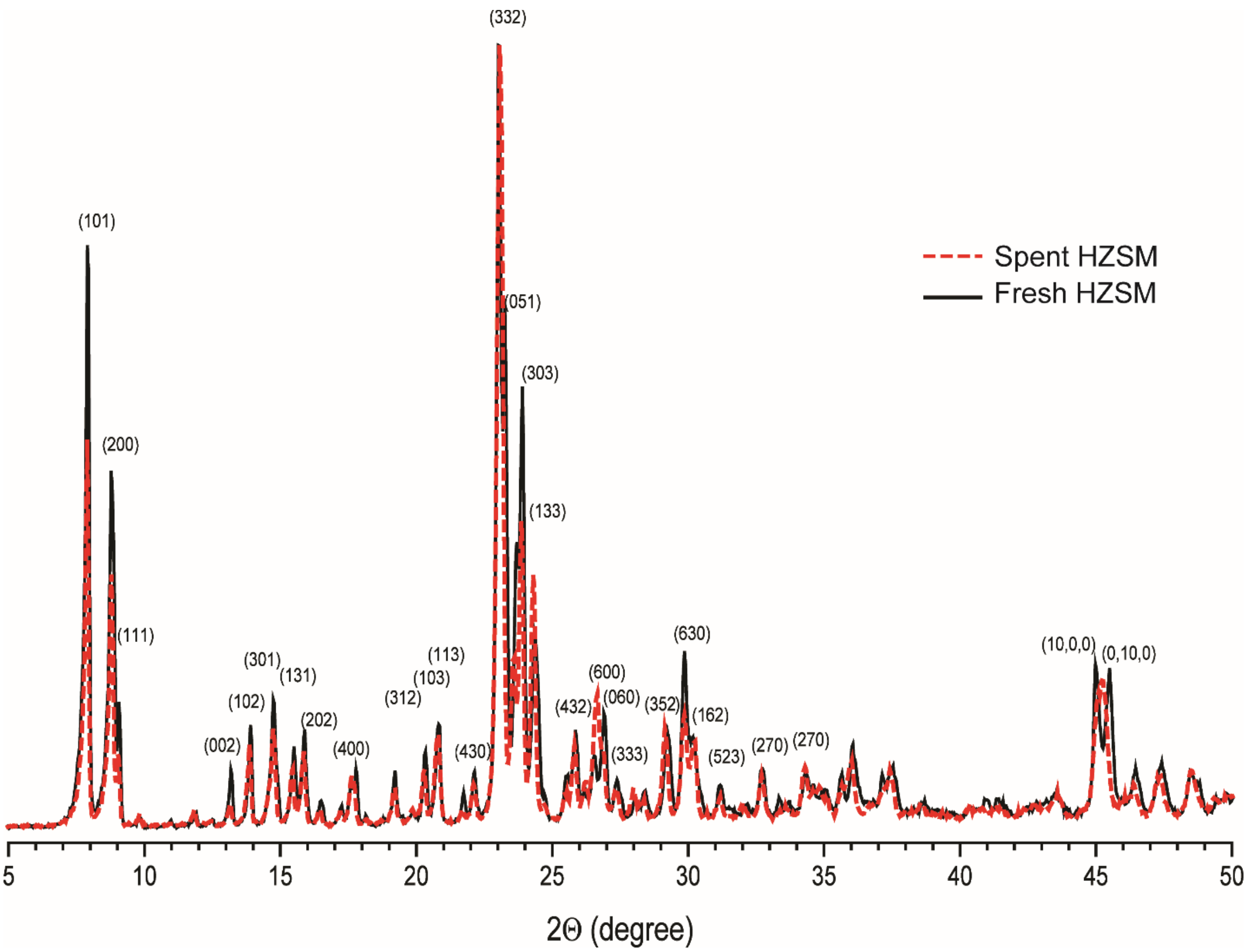

Supplementary figure 5: XRD spectra of fresh and spent HZSM-5 showing that crystallinity of the ZSM-5 did not change due to coking.

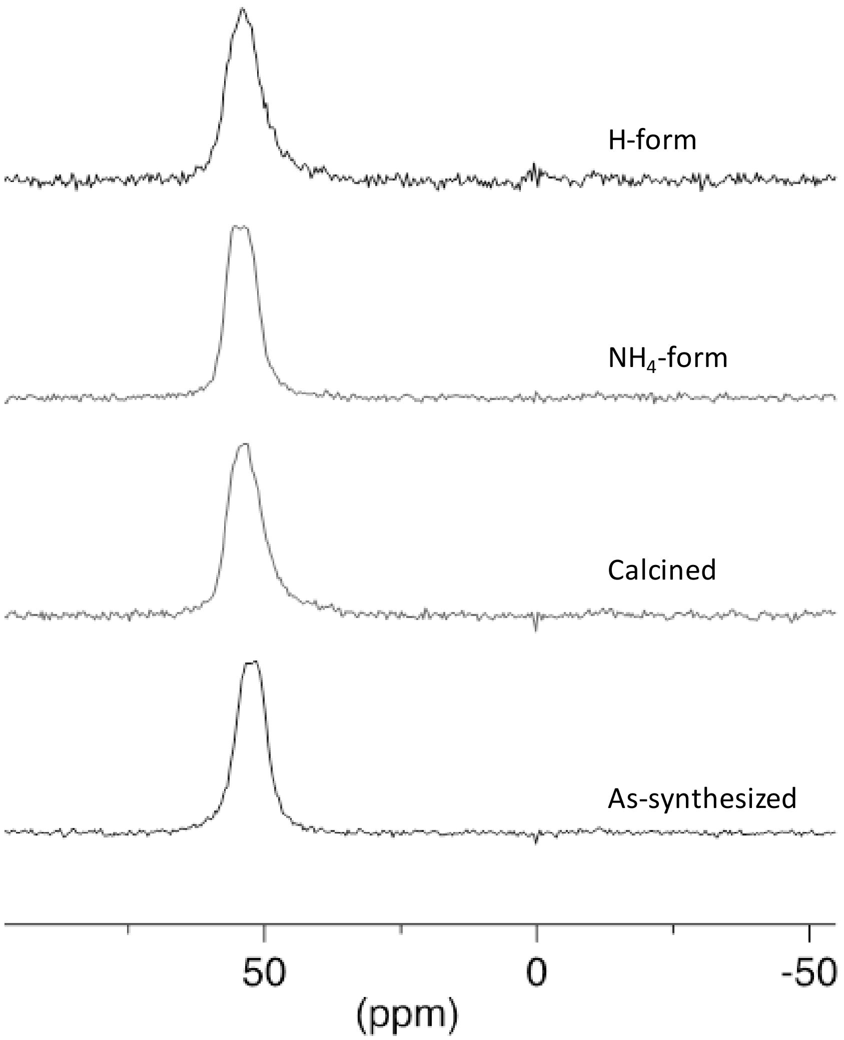

Supplementary Figure 6: ^{27}Al MAS NMR spectra of the zeolite material tested in the form of as-synthesized, calcined, NH_4 form, and H-form.
